# Multivariate genome-wide associations for immune traits in two maternal pig lines

**DOI:** 10.1186/s12864-023-09594-w

**Published:** 2023-08-28

**Authors:** Katharina Roth, Maren Julia Pröll-Cornelissen, Hubert Henne, Anne Kathrin Appel, Karl Schellander, Ernst Tholen, Christine Große-Brinkhaus

**Affiliations:** 1 Institute of Animal Science, University of Bonn, Endenicher Allee 15, 53115 Bonn, Germany; 2 BHZP GmbH, An der Wassermühle 8, 21368 Dahlenburg-Ellringen, Germany

**Keywords:** Immune traits, Pigs, Multivariate, Genome-wide Association Studies, Immunocompetence, Animal Genetics

## Abstract

**Background:**

Immune traits are considered to serve as potential biomarkers for pig’s health. Medium to high heritabilities have been observed for some of the immune traits suggesting genetic variability of these phenotypes. Consideration of previously established genetic correlations between immune traits can be used to identify pleiotropic genetic markers. Therefore, genome-wide association study (GWAS) approaches are required to explore the joint genetic foundation for health biomarkers. Usually, GWAS explores phenotypes in a univariate (uv), trait-by-trait manner. Besides two uv GWAS methods, four multivariate (mv) GWAS approaches were applied on combinations out of 22 immune traits for Landrace (LR) and Large White (LW) pig lines.

**Results:**

In total 433 (LR: 351, LW: 82) associations were identified with the uv approach implemented in PLINK and a Bayesian linear regression uv approach (BIMBAM) software. Single Nucleotide Polymorphisms (SNPs) that were identified with both uv approaches (n = 32) were mostly associated with immune traits such as haptoglobin, red blood cell characteristics and cytokines, and were located in protein-coding genes. Mv GWAS approaches detected 647 associations for different mv immune trait combinations which were summarized to 133 Quantitative Trait Loci (QTL). SNPs for different trait combinations (n = 66) were detected with more than one mv method. Most of these SNPs are associated with red blood cell related immune trait combinations. Functional annotation of these QTL revealed 453 immune-relevant protein-coding genes. With uv methods shared markers were not observed between the breeds, whereas mv approaches were able to detect two conjoint SNPs for LR and LW. Due to unmapped positions for these markers, their functional annotation was not clarified.

**Conclusions:**

This study evaluated the joint genetic background of immune traits in LR and LW piglets through the application of various uv and mv GWAS approaches. In comparison to uv methods, mv methodologies identified more significant associations, which might reflect the pleiotropic background of the immune system more accurately. In genetic research of complex traits, the SNP effects are generally small. Furthermore, one genetic variant can affect several correlated immune traits at the same time, termed pleiotropy. As mv GWAS methods consider strong dependencies among traits, the power to detect SNPs can be boosted. Both methods revealed immune-relevant potential candidate genes. Our results indicate that one single test is not able to detect all the different types of genetic effects in the most powerful manner and therefore, the methods should be applied complementary.

**Supplementary Information:**

The online version contains supplementary material available at 10.1186/s12864-023-09594-w.

## Background

In modern swine breeding, the time around birth is one main critical period for piglet survival [1, 2]. Development of breeding programs to increase general immunocompetence in order to improve piglet survival are desired. Enhancing the piglet’s immune capacity can result in further beneficial animal welfare and productivity of pigs. The immune system plays an essential role in the immunocompetence of piglets [3]. For the progress of selection strategies, basic knowledge of the genetic foundation for phenotypes associated with global immunocompetence is required.

Medium to high heritabilities (h^2^ 0.4–0.8) have been estimated for several immune traits suggesting exceeding potential of the genetic impact [4–6]. GWAS and QTL mapping can be used to explore the genetic background of immune phenotypes. Several QTL studies revealed markers throughout all chromosomes for immune traits related to red and white blood cells [7–13] as well as cytokines [14]. Previous GWAS successfully identified numerous genetic markers associated with different phenotypes such as hematological, leucocyte-related traits [15–22] and cytokines like interferone (IFN) and interleukins (IL-10) [17, 23].

Usually, GWAS addresses phenotypes in a univariate (uv) trait manner. However, a variety of multivariate (mv) methods were introduced to analyze multiple traits jointly [24]. The utilization of mv methods is recommended to increase the statistical power to detect associations [16, 25, 26]. Previous results show moderate to high genetic correlations (r_g_ 0.4–0.8) between immune traits [27]. Consideration of r_g_ between multiple immune traits can be used to identify pleiotropic genetic markers. So far, Bovo et al. [21] reported uv and mv results for the largest number of 30 hematological and clinical biochemical traits in slaughtered pigs. In these studies, pleiotropic QTL and significant tag haplotypes with effects on multiple blood parameters were detected with mv analysis e.g., a mv Bayesian approach.

The aim of this study was to identify genetic markers associated with immune traits. Besides uv GWAS the following mv statistical approaches have been applied and the results have been compared: Principal component analysis (PCA), Canonical correlation analysis (CCA), Meta-analysis (TATES) and one mv Bayesian linear regression approach (mvBIMBAM). Preliminary estimated r_g_ [27] and the construction of biological network assisted the detection of pleiotropic QTL regions. Therefore, a LR and a LW population were investigated in order to identify biologically relevant pleiotropic markers related to health and immunity.

## Results

An overview of the investigated data sets, animals and immune traits can be found in Dauben et al. [23] and Roth et al. [27]. In brief, piglets of LR and LW were phenotyped for the complete and differential blood count (15 traits), eight cytokines and haptoglobin. The experiment was conducted under mostly practical, but high hygienic conditions and without challenging the animals [23]. For the uv and mv analyses performed in this study, data sets of 522 LR and 461 LW piglets comprising 47,292 and 43,730 SNP markers, respectively, were used.

### Genetic markers identified with uv GWAS approaches

Linear and Bayesian linear regression-based approaches were applied to obtain uv GWAS results (Additional Table S1). In total 401 significant associations were identified with PLINK (LR: 324, LW: 77; adjusted p-value < 0.05). For uv BIMBAM 32 associations were detected in total (LR: 27, LW: 5; BF > 3.02). All SNPs observed with the uv Bayesian approach were also detected by the linear regression approach as implemented in PLINK. These results were mostly associated with immune traits related to red blood cells (RBC), cytokines, and haptoglobin (HAP). The identification of pleiotropic SNPs with uv GWAS is possible when genetic markers are detected across various traits. In total, 75 SNPs (PLINK: 70, BIMBAM: 5) were detected for multiple traits like RBC (RBC, HMG, HMT) and cytokines (IL1b, IL-4, IL-6, IL-10, Tumor Necrosis Factor-α (TNF)) within uv GWAS. Additionally, the uv GWAS results were compared across the investigated breeds, however, no overlapping markers were observed between the breeds (Additional Figure S1).

### Principal component analysis of the immune traits

Details of the analysis of the PCs within the breeds can be found in the study of Roth et al. [27]. In brief, within BFN red blood cells (RBC), PC1 RBC explains ~ 37% of the variation in both breeds (LR: 37.23%, LW: 37.49%). This PC is mainly influenced by RBC characteristics of haemoglobin, haematocrit and RBC. On the contrary, PC2 RBC (LR: 22.43%, LW: 22.84%) is mainly influenced by the calculated ratio of mean corpuscular haemoglobin (MCH) and mean corpuscular volume (MCV) (only in LR). Within PC3 RBC and PC4 RBC which also explain more than 10% of the variation, mean corpuscular haemoglobin concentration (MCHC) and haptoglobin are the main actors. Within the BFN cells, PC1 Cell (LW: 35.96%, LR: 35.49%) is dominated by neutrophils and lymphocytes, which were known to be negatively correlated and influenced by the time point of blood sampling. On the contrary, PC2 Cell can be characterized by the percentages of eosinophils and white blood cells (WBC) (only in LW). In BFN cytokines (Cyto), PC1 Cyto explains most of the phenotypic variation (LR: 68.13%, LW: 60.13%). This PC is similarly influenced by examined cytokines. Apart from that, the chemokines IL-12 and Il-8 have less impact on PC1 Cyto but dominate PC2 Cyto in LW piglets. PCs of the two breeds cannot be compared in general because their composition based on loading values differs from breed to breed. In contrast, we assumed that the variance components of the first PCs of each BFN (PC1 Cell, PC1 RBC and PC1 Cyto) are comparable between the breeds due to similarities in the contribution based on their loading values.

### Structural multivariate trait combinations

The identification of causal relationships among immune traits before performing mv GWAS helps to reduce extensive computation effort impaired by the realization of all possible mv combinations for all available immune phenotypes. Immune trait combinations of interest were created by performing Bayesian Network (BN) analyses based on the hill-climbing algorithm [28] for all immune traits in LR and LW data sets.

**Fig. 1:**
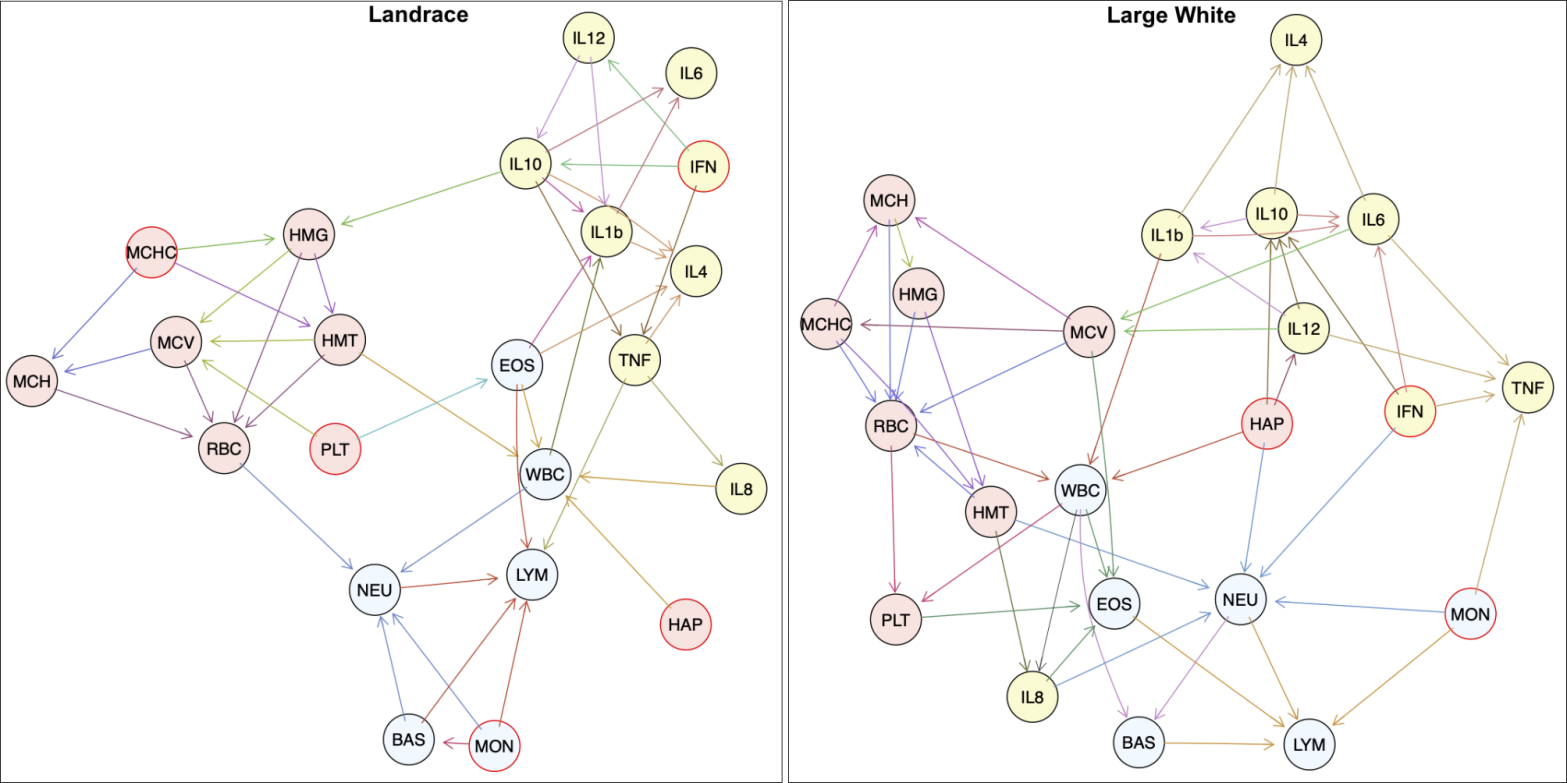
Bayesian Network for immune trait residuals RBC = Red blood cells, HMG = Hemoglobin, HMT = Hematocrit, MCV = Mean Corpuscular Volume, MCH = Mean Corpuscular Hemoglobin, MCHC = Mean Corpuscular Hemoglobin Concentration, PLT = Platelets, WBC = White blood cells, NEU = Neutrophils, LYM = Lymphocytes, MON = Monocytes, EOS = Eosinophils, BAS = Basophils, HAP = Haptoglobin, IFN = Interferon-γ, IL = Interleukin, TNF = Tumor Necrosis Factor-α. Functional biological networks of phenotypes are illustrated as nodes in pale blue for WBC, light red for RBC, and yellow for cytokines. Node frames are highlighted in red to highlight conditionally independent variables. Colored arrows are used to indicate parental relationships of the nodes in the structured model learned from the data sets

The dependencies among the variables of the structural BN model strings are illustrated in Fig. 1 and are presented in Table 1. In total 22 combinations were detected for LR and LW, respectively. In Table 1 the structure of the identified BN is displayed: a local structure is presented in square brackets [] with the first string identifying a node. There are two types of nodes: parents and children. The state input variables, or parents of the node, are listed after a vertical bar “|”, separated by colons “:“. Children of the node represent the interaction determined by the conditional probability, derived from two or more parent nodes. One trait combination [HMT|HMG:Mean Corpuscular Hemoglobin Concentration (MCHC)] was identified in both breed-specific networks allowing investigations for trait combinations within as well as across the breeds.

The causal relationships among the phenotypes are also displayed in Fig. 1. Each of the nodes (e.g. RBC, white blood cells (WBC), IL10) represents the measured phenotypes. A directed arrow from one node to another means a direct causal effect. For example, in LR, HAP has a direct causal effect on the variable WBC, which in turn affects neutrophils (NEU) and IL1B. To accentuate functional biological networks of phenotypes, nodes are illustrated in different colors. Node frames are highlighted in red when variables are conditionally independent (HAP in LW and LR, PLT in LR). Additionally, colors are used for arrows to indicate parental relationships of the nodes in the structured model learned from the data sets.

Although BNs do not serve as biological patterns, causal relationships between immune traits mostly represent biological functional subsets. Combinations mainly based of WBC, RBC, and cytokine-related clusters. The identified conditionally dependent traits by the network structure were used as mv trait combinations for mv GWAS.

**Table 1:** Resulting structural model learned from a causal network

Breed	Conditional independent	Conditional dependent with one parent	Conditional dependent with two parents	Conditional dependent with multiple parents
LR	[MCHC]	[BAS|MON]	[HMG|MCHC:IL10]	[RBC|HMG:HMT:MCV:MCH]
	[PLT]	[EOS|PLT]	[HMT|HMG:MCHC]	[MCV|HMG:HMT:PLT]
	[MON]	[IL8|TNF]	[MCH|MCV:MCHC]	[WBC|HMT:EOS:HAP:IL8]
	[HAP]	[IL12|IFN]	[IL6|IL10:IL1b]	[LYM|NEU:MON:EOS:BAS: TNF]
	[IFN]		[IL10|IFN:IL12]	[NEU|RBC:WBC:MON:BAS]
			[TNF|IFN:IL10]	[IL1b|WBC:EOS:IL10:IL12]
				[IL4|EOS:IL10:IL1b:TNF]
LW	[MON]	[IL12|HAP]	[MCV|IL12:IL6]	[RBC|HMG:HMT:MCV:MCH:MCHC]
	[HAP]	[HMG|MCH]	[HMT|HMG:MCHC]	[WBC|RBC:HAP:IL1b]
	[IFN]	[MCHC|MCV]	[MCH|MCV:MCHC]	[NEU|HMT:MON:HAP:IFN: IL8]
			[PLT|RBC:WBC]	[LYM|NEU:MON:EOS:BAS]
			[BAS|WBC:NEU]	[EOS|MCV:PLT:WBC:IL8]
			[IL1b|IL10:IL12]	[IL10|HAP:IFN:IL12]
			[IL8|HMT:WBC]	[IL4|IL10:IL1b:IL6]
				[IL6|IFN:IL10:IL1b]
				[TNF|MON:IFN:IL12:IL6]

LR = Landrace, LW = Large White, RBC = Red blood cells, HMG = Hemoglobin, HMT = Hematocrit, MCV = Mean Corpuscular Volume, MCH = Mean Corpuscular Hemoglobin, MCHC = Mean Corpuscular Hemoglobin Concentration, PLT = Platelets, WBC = White blood cells, NEU = Neutrophils, LYM = Lymphocytes, MON = Monocytes, EOS = Eosinophils, BAS = Basophils, HAP = Haptoglobin, IFN-γ = Interferon-γ, IL = Interleukin, TNF-α = Tumor Necrosis Factor-α. Conditional dependencies are indicated as straight line. Local structure is presented in square brackets [] with the first string identifying a node. Parents of the node are listed after “|” and are separated by colons “:”. Children of the node represent the interaction determined by the conditional probability, derived from two or more parent nodes. These parental relationships are also indicated in different colors for arrows in Fig. 1. The causal network model was assigned in three categories for more comprehensive understanding of the model structure. Conditionally dependent traits identified by the network structure given in [] were used as trait combinations for multivariate genome-wide association study.

### Genetic markers identified with mv GWAS approaches

Applying uv GWAS, the identification of pleiotropic genomic region is limited, especially in the situation of polygenic inherited traits. Therefore, the following four different mv approaches were applied on immune trait combinations for LR and LW in order to increase the detection power for pleiotropic SNP: PCA, CCA, TATES and mvBIMBAM. In total, 647 significant associated SNPs were detected with mv methods and can be found in the Additional Table S2.

PCA was able to detect 98 (9 genome-wide and 89 chromosome-wide significant) and 26 (5 genome-wide and 21 chromosome-wide significant) SNPs associated with the phenotypes for LR and LW, respectively.

CCA revealed a variety of associated SNPs: 416 for LR and 151 for LW. For LR, 72 were genome-wide and 344 were chromosome-wide significant. For LW, 37 were genome-wide and 144 were chromosome-wide significant.

Twenty-eight genome-wide significant markers were determined with TATES for LR while 3 genome-wide significant genetic variants were characterized as significant for LW.

mvBIMBAM detected 8 and 23 genome-wide significant SNPs for LR and LW, respectively.

All detected SNPs with mv methods were summarized to 190 QTLs, by assuming a 1 Mbp interval around significant SNPs. Out of these QTLs, 133 were located within or close located to protein-coding genes. Functional annotation of these QTLs revealed 453 protein-coding genes (Additional Table S2).

### Comparison across mv GWAS results

**Fig. 2:**
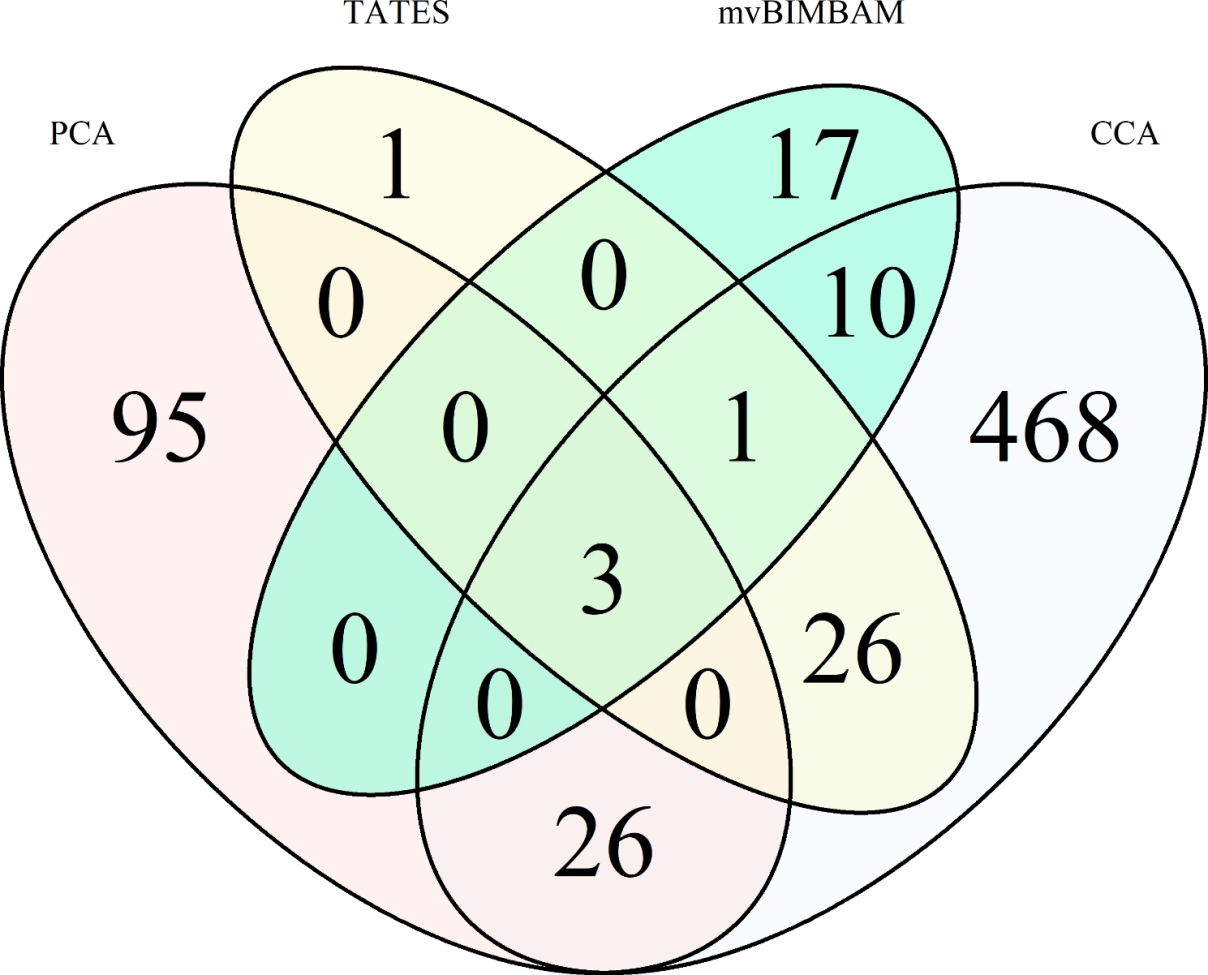
Venn diagram of different methods used to detect significant multivariate associations summerized for both breeds and significance types PCA = Principal component analysis, CCA = Canonical correlation analysis, TATES = Trait-based Association Test that uses Extended Simes procedure, mvBIMBAM = multivariate Bayesian IMputation-Based Association Mapping. Multiple identical significant SNPs for different immune traits within a method are counted once

SNPs that are identified with multiple mv methods are of particular interest to characterizing pleiotropy. In total, 66 SNPs were detected for different trait combinations with more than one mv method (Fig. 2). Thirty-seven of these SNPs are associated with RBC related immune trait combinations (e.g. [RBC|HMG:HMT:Mean Corpuscular Volume (MCV):Mean Corpuscular Hemoglobin (MCH), HMG|MCHC:IL10, HMT|HMG:MCHC]. Thirteen SNPs are associated with WBC subtypes and 12 with cytokines. For example, SNP ALGA0073579 (rs81442304) was identified with three mv methods CCA, TATES, as well as mvBIMBAM. CCA and TATES associated this SNP with BAS|MON in LR, whereas mvBIMBAM detected this association for cytokines IL-4|IL-10:IL-1b:IL-6 in LW. Currently, this SNP remains unmapped for *Sscrofa* 11.1. SNPs ALGA0086892 (rs81454413, SSC 15: 116.13 Mbp), ASGA0070586 (rs80818610, SSC15: 120.11 Mbp), and ASGA0070620 (rs80883544, SSC 15: 120.35 Mbp) were detected by all four mv methods in LR for cytokines and a five immune trait combination of WBC, HMT, eosinophils (EOS), HAP, and IL-8. With PCA these SNPs were observed for the second PC in the biological functional network of cytokines (PC2 Cyto). According to the contribution based on loading values, this PC mainly contains cytokines IL-12 and IL-8 [27]. These SNPs are located on *SSC* 15 within an intron region of four Mbp (116.13 to 120.35 Mbp) (Fig. 2; Table 2).

**Table 2:** Selected significant associated genetic markers identified with multivariate methods

Breed	Trait	SSC	SNP	Pos	m/M	MAF	p-value/BF	Method	Gene
LR; LW	BAS|MON, NEU|RBC:WBC:MON:BAS; IL-4|IL-10:IL-1b:IL-6	13*	ALGA0073579	203.4*	T/C	0.01 and 0.21	0.01/3.22	CCA, TATES, mvBIMBAM	
LR; LW	HMG|MCHC:IL-10, PC4Cell; PLT|RBC:WBC	5*	H3GA0016899	80.1*	T/C	0.04 and 0.16	0.04	CCA, PCA	
LR	IL-8|TNF, WBC|HMT:EOS:HAP:IL-8, PC2Cyto	15	ALGA0086892	120.1	T/C	0.50	0.04/3.5	CCA, PCA, TATES, mvBIMBAM	SPAG16
LR	IL-8|TNF, WBC|HMT:EOS:HAP:IL-8, PC2Cyto	15	ASGA0070586	120.1	T/C	0.41	0.01/4.77	CCA, PCA, TATES, mvBIMBAM	TNS1, RUFY4, CXCR2, ARPC2, GPBAR1, AAMP, PNKD, TMBIM6
LR	IL8|TNF, WBC|HMT:EOS:HAP:IL-8, PC2Cyto	15	ASGA0070620	120.3	T/C	0.39	0.03/4.04	CCA, PCA, TATES, mvBIMBAM	

SSC = *Sus scrofa* chromosome, SNP = single nucleotide polymorphism, Pos = Position [Mbp] m/M allele = minor/major allele, MAF = minor allele frequency, p-value = adjusted p-value after correction for stratification and multiple testing, BF = Bayesian factor, Gene = selected nearest gene within a progressive number of QTL based on ± 1Mbp distance from a significant SNP, LR = Landrace, LW = Large White, BAS = Basophils, MON = Monocytes, IL = Interleukin, HMG = Hemoglobin, HMT = Hematocrit, NEU = Neutrophils, RBC = Red blood cells, WBC = White blood cells, PLT = Platelets, IFN = Interferon-γ, TNF = Tumor Necrosis Factor-α, PC = Principal component, Cell/Cyto = Biological functional networks within the PCA cell/cytokines, PCA = Principal component analysis, CCA = Canonical correlation analysis, TATES = Trait-based Association Test that uses Extended Simes procedure, mvBIMBAM = multivariate Bayesian IMputation-Based Association Mapping

* genome positions according to the assembly SScrofa 10.2

**Fig. 3:**
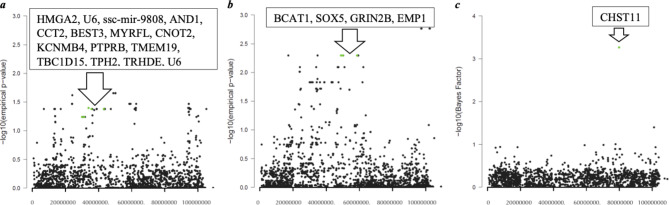
Manhattan plot of *SSC* 5 for multivariate trait combinations *a* RBC|HMG: HMT:MCV:MCH in Landrace with CCA, *b* HMG|MCHC:IL10 in Landrace with CCA, and *c* WBC|RBC:HAP:IL1b in Large White with mvBIMBAM RBC = Red blood cells, HMG = Hemoglobin, HMT = Hematocrit, MCV = Mean Corpuscular Volume, MCH = Mean Corpuscular Hemoglobin, MCHC = Mean Corpuscular Hemoglobin Concentration, IL = Interleukin, WBC = White blood cells, HAP = Haptoglobin, SNPs of interest are highlighted with green color (**a** DRGA0005609 (rs80847233), ASGA0025326 (rs80801793, SSC 5: 31.27 Mbp), ALGA0031690 (rs80785563, *SSC* 5: 33.95 Mbp), MARC0021861 (rs80948498), DRGA0005776 (rs336848545, *SSC* 5: 43.22 Mbp), **b** ALGA0031924 (rs80949260, *SSC* 5: 48.90 Mbp), MARC0001027 (rs81284886, *SSC* 5: 50.09 Mbp), ALGA0032074 (rs80787531, *SSC* 5: 58.60 Mbp), and **c** MARC0013873 (rs80911910)). Protein coding genes within annotated QTLs between 23.93 and 97.48 Mbp are stated in the box

In addition, 152 markers were identified for multiple mv trait combinations (Additional Table S2). Identical SNPs were mostly shared between immune traits related to functional biological immune trait subsets like RBC (e.g. [HMT|HMG:MCHC], MCH|MCV:MCHC], [RBC|HMG:HMT:MCV:MCH]), WBC subtypes (e.g. [NEU|RBC:WBC:Monocytes (MON):Basophils (BAS)], [Lymphocytes (LYM)|NEU:MON: EOS:BAS:TNF]) and cytokines (e.g. [IL1b|IL10:IL12], [IL4|IL10:IL1b:IL6], [IL6|IFN:IL10:IL1b]). These markers are distributed over all 18 chromosomes. Interestingly, 30% of identical markers are located on *SSC* 5 between 23.93 and 97.48 Mbp and cover 16 QTLs including 20 protein-coding genes (Fig. 3).

In addition, mv results were compared across the investigated breeds. In total, 469 markers were identified for LR, whereas 180 were detected for LW applying mv GWAS. Two SNPs, ALGA0073579 (rs81442304) and H3GA0016899 (rs80959576), were repeatedly observed in both breeds (Table 2). These markers were identified by applying mv methods (CCA, TATES, mvBIMBAM) as well as with uv methods.

### Comparison between uv and mv GWAS results

**Fig. 4:**
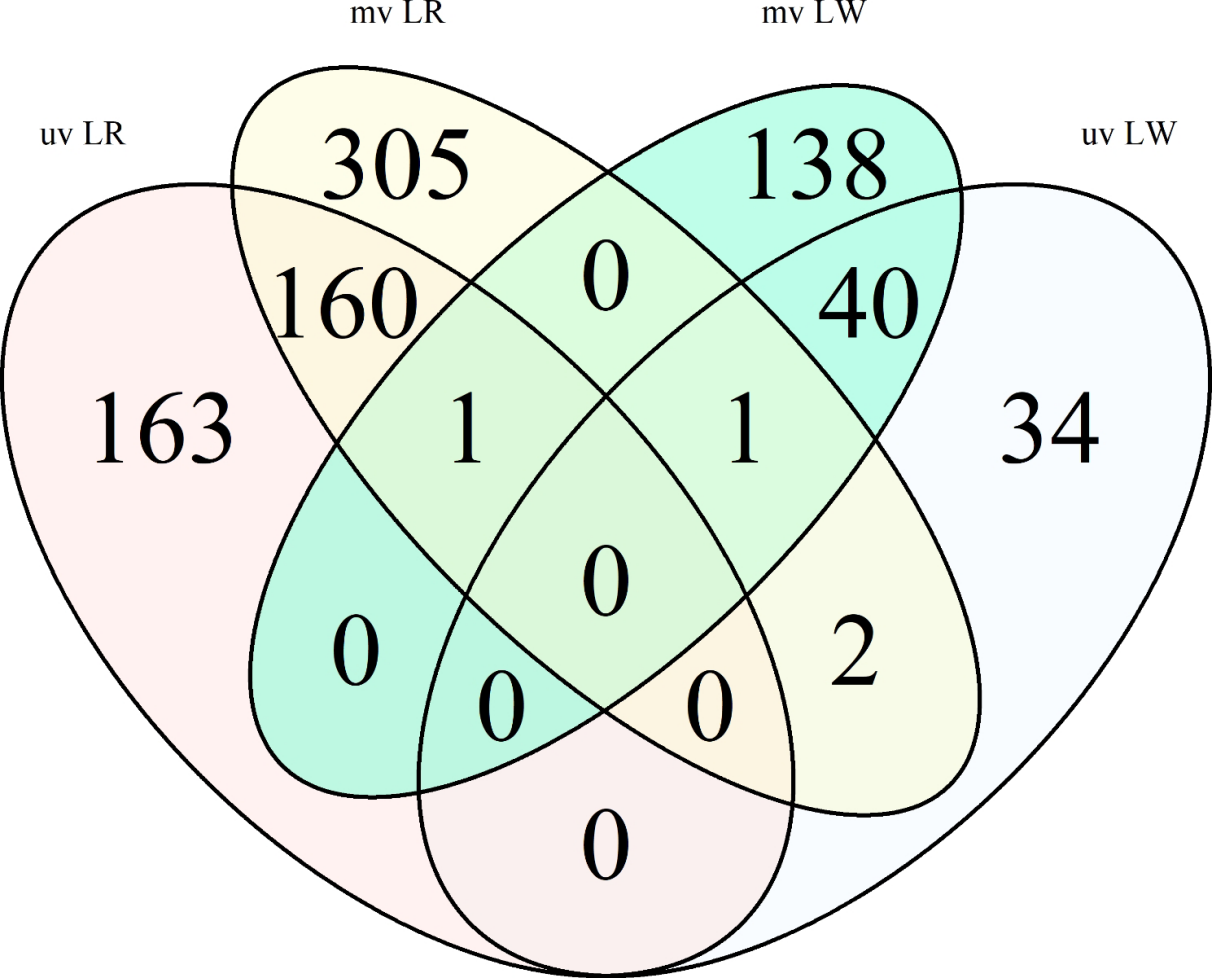
Genetic markers identified with GWAS approaches: Comparison of different association methods for both investigated breeds Multiple identical significant SNPs for different immune traits within a method are counted a single time. mv = multivariate, uv = univariate, LR = Landrace, LW = Large White

In addition, a comparison of the uv and mv results revealed that 204 markers overlap across the methods (Fig. 4). All in all, these 204 markers are located near 125 protein coding genes. Filtering the overlapping SNPs for the investigated breeds revealed four interesting genetic variants (ALGA0073579 (rs81442304), H3GA0016899 (rs80959576), DRGA0006061 (rs81303269, *SSC* 5: 79.02 Mbp), ALGA0113815 (rs81342648)) that overlap between uv and mv methods (Fig. 4).

CCA revealed, that ALGA0073579 (rs81442304) was significantly associated with [BAS|MON] in LR, whereas, applying mvBIMBAM, this SNPs was observed for cytokines [IL-4|IL-10:IL-1b:IL-6] in LW. Additionally, this SNP was also identified for the trait basophils in LR within uv GWAS using PLINK.

H3GA0016899 (rs80959576) was significantly associated with PC4 Cell in LR. According to the loading value, PLT and HAP mostly contributed to PC4 Cell. Applying CCA allowed to detect this SNP for [PLT|RBC:WBC] in LW. Furthermore, H3GA0016899 was also significantly associated with RBC in LW using an uv GWAS.

The genetic variant DRGA0006061 (rs81303269, *SSC* 5: 79.02 Mbp) was identified for [IL4|EOS:IL10:IL1b:TNF] with CCA in LR, whereas PLINK detected this association for RBC in LW. Currently, the SNP H3GA0016899 is unmapped for *Sscrofa* 11.1, but was previously mapped on SSC 5.

On *SSC* 12, within and intron region of the Regulator of G-protein signalling 9 (RSG9) gene (12.0 Mbp), the SNP ALGA0113815 (rs81342648) was significantly associated with a PC2 Cyto (consisting of cytokines IFN-γ, IL-12, IL-8 specified by the loading value) by applying the PCA approach in LR, whereas PLINK identified this association for IL-4 in LW (Additional Tables S1 and S2).

## Discussion

The aim of this study was the detection of genetic markers associated with immune traits applying different approaches of uv and mv GWAS. In total, 401 and 647 significant associations were identified with uv GWAS and mv GWAS, respectively. Of particular interest are the created immune networks using BN and PC analyses.

### Conditional dependencies of immune networks

For mv analysis, 22 available immune phenotypes would result in multiple possible mv combinations, which would require high computational effort. The application of a BN approach allowed to identify conditional dependencies among immune traits and to focus on relevant trait combinations. Usually, BNs do not reflect biological patterns when causal statistical relationships between variables have been detected. However, identified combinations can be classified into biological functional subsets of immune traits. For both pig lines, conditional relationships were identified within RBC-related traits, WBC subtypes, and cytokines. These networks correspond to previous estimated r_g_ results [27]. RBC were highly correlated with RBC characteristics, like HMT (LR: 0.82 ± 0.05, LW: 0.90 ± 0.09) and HMG (LR: 0.81 ± 0.06, LW: 0.77 ± 0.10). As expected, among further RBC characteristics, a high positive correlation was found between HMT and HMG (LR: 0.99 ± 0.00, LW: 0.97 ± 0.04), MCH, and MCV (LR: 0.99 ± 0.02, LW: 0.94 ± 0.03). Between cytokines such as IFN-γ, IL-10, IL-1β, IL-4, and IL-6 high positive r_g_ were estimated in both investigated pig lines. Immune cells such as MON and EOS were positively correlated to cytokines like TNF-α in LR but showed a high negative correlation in LW. Ballester et al. [22] constructed a network based on phenotypic correlations for immune traits in Duroc piglets. Although only 13 immune parameters overlap between Ballester et al. [22] and our study, similar clusters that relied on RBC and WBC subtypes were identified. The detected close relationships in the previous and current studies [22, 23, 27, 29] indicate the complexity of piglet’s immunity.

As discussed by Roth et al. [27] the PCA aims for a more powerful analysis of the immune traits by reducing the dimension of information, and therefore allowing the detection of key players in immunocompetence. In that study, PCA was shown to be an effective tool for condensing information based on a phenotypic covariance matrix. Using such a technique can reduce the number of dependent variables without compromising important information [30]. Furthermore, PCAs provide an appropriate weighting of individual traits. In general, all observed phenotypic and genetic correlations as well as conditional dependencies among immune parameters, might be helpful to create well-balanced breeding selection strategies to improve the immunocompetence of pigs.

### Comparison between uv and mv GWAS results and method performance

Beside one uv frequentist and one uv Bayesian approach, four mv approaches (PCA, CCA, meta-analysis, mv Bayesian linear regression) were applied on two maternal pig lines. Results were empirically compared within and across the methods.

Comparing the uv approaches, identical significant associations were detected. The investigated data sets were also studied by Dauben et al. [23] using the GenABEL-package in R [31] and ASReml Software [32]. In total, Dauben et al. [23] identified 25 genome-wide and 452 chromosome-wide significant SNPs (LR: 280, LW: 197) associated with 17 immune relevant traits in both pig lines. Applying PLINK and uvBIMBAM it was possible to identify 433 (LR: 351, LW: 82) significant associations. Comparing the results of both studies, 159 and 15 associations were commonly detected for LR and LW, respectively.

One reason for the different number of significant SNP markers among the studies are caused by the requirement for the multivariate analyses. The number of phenotypes per animals have to be complete. Furthermore, the applied methods to correct for false positives and the determined threshold for genome-wide and chromosome-wide significance differ depending on the applied methodology.

Among the common associations in LR, 49 SNPs were also identified with mv methods in this study. Common results were mostly associated with immune traits related to RBC, cytokines, and HAP e.g. ASGA0070620 (rs80883544, *SSC* 15: 120.35 Mbp). The SNP ASGA0070620 is located near protein-coding genes such as TMBIM1 (transmembrane BAX inhibitor motif containing 1).

Generally, previous GWAS studies for immune traits focused mostly on uv statistical approaches. The application of mv methods is recommended to increase the statistical power to detect associations [16, 21] even if the r_g_ between the traits is expected to be weak (close to 0) [25, 26]. Consideration of previously published high r_g_ ( ≥ ± 0.4) results between multiple immune traits [27] was used to increase GWAS power to identify pleiotropic SNPs. In this study, mv methods revealed a higher number of significant associations compared to uv methods. Moreover, there was a substantial overlap of associations found by several mv methods which have different underlying statistical backgrounds. These results could be used as heuristic arguments, that mv-methods have a higher detection power. However, it should be considered that the number of approaches differs between the applied methods. For uv analysis, two different approaches were compared, whereas for mv analysis four different mv methods were utilized.

204 SNPs were identified with uv and mv methods. When SNPs are detected with multiple approaches, they provide more certainty for the GWAS results and contribute to potential candidate genes. However, 443 associations were exclusively identified with mv approaches. This underlines the importance of considering the correlation among immune traits with mv methods. Common markers for comparable trait complexes were also identified between different mv approaches. Nevertheless, markers match incompletely and only to a small extent.

Application and comparison between multiple uv and mv approaches were addressed mostly on simulated data [25, 26], rather than on immune phenotypes. Recently, Bovo et al. [21, 29] reported uv and mv GWAS results for hematological and blood clinical-biochemical traits in LW pigs after slaughtering. Similarly, to our study, one frequentist and one Bayesian approach were applied. In general, the performance of different mv approaches is scenario-specific and sensitive to specific effects like allele frequency, the number of investigated traits, and underlying correlation structures among the traits [25, 26]. Galesloot et al. [25] concluded that mv methods implemented in software like PLINK, SNPTEST, MultiPhen, and mvBIMBAM performed best in terms of detection power for the majority of scenarios, which is partly consistent with our results.

Furthermore, it has to be mention, that the possibility of chromosome-wide correction for multiple testing was not applied in every approach and was limited to methodology implemented in PLINK and R. For CCA, the highest number of associated SNPs was reported in our analysis. Similar to our results, Galesloot et al. [25] studied high power for almost all scenarios for the same approach. These authors explain higher power was observed with increasing residual correlation in case of a single QTL trait and when two or all three traits were associated with the QTL with a negative genetic correlation for methods including CCA. Due to trait correlations, test statistic distributions are likely to have longer tails, and therefore a more conservative threshold is recommended to maintain the type I error at 5% [25]. As recommended by Galesloot et al. [25], we lowered the threshold within the CCA approach (5% default value to 1% lowered threshold) and compared the association results empirically once again (results not shown). The number of detected SNPs with CCA lowered to 184 (LR: 144, LW: 40). However, the common three SNPs, which were detected with all four mv approaches, remained in the results for CCA after lowering the threshold.

Zhou et al. [33] developed an efficient linear mixed model algorithm for GWAS which is implemented in the software GEMMA and compared this algorithm to those implemented in WOMBAT [34] and GCTA [35]. Algorithms were applied to different numbers of phenotypes in simulated data as well as human and mouse data sets. Even though the authors reported exceeded improvements in computational time and power, they recommended considering the methods as complementary rather than competing. One single test is not able to detect all the many different types of genetic effects in the most powerful manner. Salinas et al. [36] described many of the mv methods aimed to detect genetic pleiotropy in an epidemiological context. In their study, specific method selection considering phenotype distribution type and data availability was developed. Therefore, our results contribute to a deeper understanding of the performance power and selection of suitable mv methods.

### Comparison of genetic markers between LR and LW

A comparison of results regarding breed differences was realized since GWAS methods were applied to the investigated breeds separately. With uv methods, no overlapping markers were observed, whereas mv methods were able to identify two SNPs shared between LR and LW. These two significant SNPs were currently unmapped. Using the older assembly 10.2 H3GA0016899 (rs80959576) was located on SSC5 (80.17 Mbp) as an intergenic variant and ALGA0073579 (rs81442304) on SSC13 (203.44 Mbp) within the GRIK1 gene, which function has not been described so far. Thirty-eight SNP listed in Table 2 could not be allocated by current assembly *SScrofa* 11.1 but were mapped under *SScrofa* 10.2. Therefore, these results should be considered with caution.

Several GWAS and QTL studies for immune competence traits investigated cross-bred (White Duroc x Erthulin F2, LR x Duroc x Yorkshire, LW x Minzhu F2) and pure-bred (Chinese Sutai, LR, LW, Songliao Black, Yorkshire) pigs [7–21, 23]. Even though the results of these studies reported a few overlapping QTL regions, most of the markers were not shared between the studies. Genetic heterogeneity of the investigated pig populations, differences in the analyzed immune traits, variety of the experimental designs, and therefore, different environmental effects considered in the statistical models during the analysis, might explain the discrepancies among the studies and between the breeds. In the current study, further options for pre-selection of the breed-specific mv trait combinations can be applied to enable appropriate comparison between the breeds within mv methods.

### Identification of potential pleiotropic genetic variants

When a locus influences several traits at the same time, pleiotropy is responsible for genetic and phenotypic correlations [37]. Human complex traits have been extensively reviewed and discussed under different definitions of cross-phenotype association (biological, mediated, spurious) (e.g. [38, 39].). However, in a joint analysis of complex traits, autocorrelations suggest pleiotropic effects.

The mv GWAS provides a higher level of precision and detection power in mapping pleiotropic QTL than uv analyses [40–43]. In particular, this applies when studying traits that are highly correlated or when heritability is low for the trait affected by the QTL [43]. Nevertheless, correlated traits may lead to correlated sampling errors [44]. A PC method has been described as a more powerful alternative to a single trait analysis [45, 46]. This approach condenses traits of interest into a number of uncorrelated PCs that reflect the underlying (co)variance matrix. According to Mähler et al. [47], it has been suggested to analyze only the first PC since it explains the majority of the variation. It has been demonstrated that the second PC and subsequent PCs can identify the highest phenotypic proportion that can be explained by genetic markers [48]. According to the authors, the second and following PCs may contain a substantial proportion of total genetic variation, which normally accounts for a small amount of variance in phenotypic traits. If the QTL effects oppose positively correlated traits, these PCs appear very powerful.

Using the first three PCs, this study determined that a significant portion of the total genetic association could be attributed to these PCs. However, genetic interpretation of the identified association is impossible with this approach, despite higher statistical power. Due to unclear pleiotropy or high linkage between two regions, there is not yet a clear indication of true pleiotropy [40]. This analysis is generally considered a first step in identifying pleiotropic regions, which would require further investigation with more precise models, fine-mapping or molecular experiments to confidently distinguish between the different scenarios.

### Functional annotation and identification of potential candidate genes

Using different uv and mv GWAS approaches in this study it was possible to detect a plethora of genetic markers. SNPs were summarized into QTLs, based on their genetic distance of 1 Mbp downstream and upstream, to condense functional information. Annotation was performed within the characterized QTLs in *Sscrofa* 11.1 from the Ensembl database [49]. QTLs were located within numerous protein-coding genes (uv: 354, mv: 453). 125 protein-coding genes were identified with both methods (uv and mv) and selected immune relevant genes are presented in Table 2 and Table S1 and S2. The SNP ASGA0070586 (rs80818610, *SSC* 15: 120.11 Mbp), located on *SSC* 15, was detected applying all four multivariate approaches. In the following, three out of eight candidate genes are discussed. AAMP (angio associated migratory cell protein) plays a positive role in angiogenesis, a physiological process through which new blood vessels are formed from pre-existing vessels [50]. PNDK (paroxysmal nonkinesiogenic dyskinesia domain containing) protein is involved in the regulation of neurotransmitter secretion and is associated with pancreatic, ovarian, and breast cancer in humans [51, 52]. In swine, a disruption of expression and pathway of PDNK in response to infection with *Actinobacillus pleuropneumoniae* bacteria was observed [53]. TMBIM1 (transmembrane BAX inhibitor motif containing 1) protein binds to a TNF receptor and thus regulates the degranulation of neutrophils and the reorganization of blood vessels [54]. Five additional gene were located close to ASGA0070586 (rs80818610, *SSC* 15: 120.11 Mbp), but a functional immune relevant relationship have not been described yet.

On *SSC* 14 the marker MARC0013023 (rs80797218) was significantly associated for HMG and HMT using uv PLINK and BIMBAM. In addition, this SNP was also detected applying CCA for the traits HMT, HMG and MCHC applying CCA. Within this region the protein-coding gene AGT (angiotensinogen) is located, that regulates the systemic arterial blood pressure by renin-angiotensin [55]. According to their direct influence on immune traits these protein-coding genes represent potential candidate genes.

Some of the genetic markers detected in this study have been identified in previous association studies for hematological traits. Wang et al. [16] detected SNPs ALGA0123028 (rs81318039, *SSC* 3: 71.12 Mbp) and MARC0001946 (rs81288717, *SSC* 3: 72.97 Mbp) located on *SSC* 3 for mean thrombocyte volume. These SNPs were identified for immune trait combination [WBC|HMT:EOS:HAP:IL8] in LR. In the study of Lu et al. [17] MARC0039159 (rs81232385, *SSC* 5: 44.44 Mbp), located on *SSC* 5, was significantly associated with IL-10, which was identified in our study with CCA and PCA for [NEU|RBC:WBC:MON:BAS] and PC3 Cell (LYM, MON, BAS contribute to this PC according to the loading value), respectively. Luo et al. [15] identified ALGA0047813 (rs81400288, *SSC* 8: 43.03 Mbp) and MARC0039159 (rs81232385, *SSC* 5: 44.44 Mbp) on *SSC* 8 for MCV and MCH, which was observed in our study for the mv trait combination [RBC|HMG:HMT:MCV:MCH:MCHC] in LW with CCA. ALGA0047813 (rs81400288, *SSC* 8: 43.03 Mbp), is located within the intron region of the protein-coding gene TLL1 (tolloid like 1). Studies in mice suggest that TLL1 plays multiple roles in the development of the mammalian heart, and is essential for the formation of the interventricular septum. Allelic variants of this gene are associated with atrial septal defect type 6 [56]. Further investigations of this protein function in pigs are needed, to determine the potential as a candidate gene. Dauben et al. [23] detected associations for immune traits in the same pig population with a different uv GWAS approach. Identical markers have been identified between this and the current study (LR: 159, LW:15). Noteworthy, 49 SNPs identified in LR were observed with uv and mv methods. Therefore, in this study we were able to confirm associations with our previous results.

## Conclusion

This study evaluated the joint genetic background of immune traits in LR and LW piglets through the application of various uv and mv GWAS approaches. In general, mv GWAS approaches outperformed uv methods and detected genome-wide associations for immune traits. It should be considered that the number of significant associations differs between the applied methods and the possibility of chromosome-wide correction for multiple testing was only feasible in two approaches. When associations were compared across the investigated breeds, no overlapping markers were observed with uv methods, indicating genetic breed differences. It was possible to detect two SNPs in both breeds applying mv GWAS. However, further options for pre-selection of the breed-specific mv trait combinations and cross-validation should be considered to enable appropriate breed comparison. Our results support the observation that one single test is not able to detect all the many different types of genetic effects in the most powerful manner. These analyses are initial steps to detect pleiotropic regions in general. Beside the validation of our results with other data sets, it is necessary investigate the identified associations further applying fine-mapping approaches and the analyses of candidate genes.

### Methods

#### Statistical analysis of immune traits

Data sets of purebred LR and LW populations were recorded from 2010 to 2017 and were provided by the German breeding organization BHZP GmbH. Animal care, phenotypic measurements, and consideration of environmental effects were described in Roth et al. [27]. In brief, a total of 611 piglets (♂152/♀307) of LR and 533 piglets (♂134/♀257) of LW were analysed. Animals were a subset of two nucleus populations. From each litter, one male and one female piglet, were chosen for phenotype collection. Blood samples of piglets were collected on average around 45 days (32– 60) after birth by puncturing the Vena jugularis and were collected in three 7.5 ml monovette containing ethylenediaminetetraacetic acid. Complete blood count was performed with an ADVIA® 2120 Hematology system, a flow cytometry- based system, and a pig- specific setting. Besides, serum haptoglobin was measured in 0.5 ml serum. Peroxidase activity of the haptoglobin– haemoglobin complex was carried out by a spectrophotometric method. Cytokine levels (interferon- γ, interleukin- 10, interleukin- 12, interleukin- 1β, interleukin- 4, interleukin- 6, interleukin- 8 and tumour necrosis factor- α) in serum samples were analysed with a Porcine Cytokine/Chemokine Multiplex Magnetic Bead Panel (Merck KGaA) enabling the simultaneous measurement of multiple cytokines. Immunoassay of serum samples was performed using 22 plates according to the manufacturer´s protocol.

GWAS was performed for complete blood count (RBC, haemoglobin, haematocrit, MCV, MCH, MCHC, platelets, WBC, neutrophils, lymphocytes, monocytes, eosinophils, basophils, band and other remaining cells), HAP, and cytokines (interferon-γ, interleukin-10, interleukin-12, interleukin-1β, interleukin-4, interleukin-6, interleukin-8 and tumour necrosis factor-α) as immune traits of 1144 LR and LW piglets, corrected for environmental impacts within the breeds. A detailed description of all investigated immune traits, their summary statistics, and processing of the data set can be found in Roth et al. [27].

### Genotyping and quality control of genomic markers

To study genetic associations between measured phenotypes animals were genotyped with a tissue sample via an Ilumina Porcine SNP60 v2 BeadChip (Illumina, San Diego, CA, USA) in an external laboratory (GeneControl GmbH, Poing). Only autosomal markers were used in the different GWAS approaches. Regardless of the selected association method, quality control of genotype data was performed with PLINK [57]. Genetic markers and animals were excluded when they did not meet the following criteria: Call Rate ≥ 0.95, Minor allele frequency (MAF) ≤ 0.01, deviation from Hardy-Weinberg equilibrium (HWE) p-value = 0.0001, acceptable Identity-by-state (IBS) threshold ≤ 0.95. After quality control 47’292 and 43’730 markers, as well as 522 and 461 animals, remained for GWAS for LR and LW, respectively. The position in the genome and the base pair location of each SNP is based on *SScrofa* 11.1. In total, 38 markers show currently no location under this assembly. Using the assembly *SScrofa* 10.2 it was possible to report a chromosome number and a base pair position for 15 markers. The remaining 22 markers revealed high linkage disequilibrium to other significantly associated SNP (results not shown). The observed regions correspond to the positional information given in the manifest file of the manufacturer.

### Correction for environmental effects

The correction for environmental effects was performed within a breed and included all relevant fixed effects: the class effects parity (1–4) and herd-year-season-sex (1–12). Moreover, age and weight and interaction between age and weight at the time of sample collection were included in the model as covariates. Cytokine detection method requires the quantification of samples distributed among 22 analytical plates. Therefore, plate was included as a random term for cytokine immune traits. The effects of breed (LR or LW) or sex (boar or sow) were not included as main factors in the model because of the hierarchical classification of these effects within herd-year-season-sex classes.

### Univariate GWAS

After quality control, one frequentist and one Bayesian method were used to analyze immune traits for uv associations with the genotype in a GWAS within each breed data set.

The starting point for both approaches is a mixed linear model:

1 $$y=\mu +Z\alpha +e$$

where $y$ is a vector of phenotype measurement of animals, $\mu$ is a vector of the phenotype means of animals carrying the reference genotype, Z is a matrix of genotype covariates (coded as 0, 1, or 2) for SNP markers, $\alpha$ is a vector of random regression coefficients of the SNPs (marker effects), and $e$ is a vector of residuals.

The frequentist association approach in PLINK [57] tests each marker for association with the trait of interest since it performs a linear regression analysis with each SNP as a predictor. For Bayesian regression, prior distributions are specified for $\alpha$ and$e$. For vector of residuals $e$, a prior conditional on the residual variance, ${\sigma }_{e}^{2}$, a normal distribution with null mean and covariance matrix ${R\sigma }_{e}^{2}$, is used. In this case, $R$ is a diagonal matrix and ${\sigma }_{e}^{2}$ is treated as an unknown with a scaled inverse ${\chi }^{2}$ prior [58]. Assuming that a SNP $j$ is a Quantitative Trait Locus, then its effect is dependent on two parameters: ${a}_{j}$ and ${d}_{j}={a}_{j}{k}_{j}$: the additive and dominance effect, respectively. An additive effect is given by ${k}_{j}=0,$ while ${k}_{j}=1$ and ${k}_{j}=-1$ represents a dominant effect. Bayesian linear regression carried out with BIMBAM uses two priors D_1_ and D_2_ to model this effects [59]. Bayesian Factors for observed associations were computed as posterior distributions for SNP effects using the prior D_2_ averaging ${a}_{j}=\text{0.05,0.1,0.2,0.4}$ and ${d}_{j}={a}_{j}/4$. Further detailed information about the utilized uv GWAS approaches can be found in the original literature [57–59].

### Principal component analysis

To condensate the estimated highly correlated immune network PCA was applied to immune observation residuals. PCA proceedings steps and results are already published and described in detail in Roth et al. [27]. Before the application of the PCA technique for each breed data set, we split the immune traits of our survey into three biological functional networks as (a) Cells, (b) RBC (including HAP) and (c) Cytokines. This classification was motivated by the strategy to maintain the greatest possible explained variance from the original variables in the constructed PCs. The number of PCs used to characterize immune traits was based on the eigenvalues of their correlation matrix. In order to limit the number of PCs, PCs with eigenvalues lower than 1.0 were excluded [60]. As far as possible, loading values of PCs were used to label them roughly and to interpret PCs according to their summarizing biological composition. BFN-specific PCs were then used as new traits during a uv GWAS which was carried out with PLINK [57]. The output of the association analysis generates an asymptotic significance value (p-value). Received p-values were adjusted for population stratification and multiple testing on genome and chromosome levels.

#### Learning structures using bayesian network

The realization of all possible mv combinations for all available immune phenotypes is computationally extensive. Networks, paths, and graphs can model interactivity between variables. BN describe conditional in- and dependence relationships among variables [61]. Therefore, in this study, a BN approach was performed for each breed data set to reveal conditional dependencies among immune traits. Applying this approach, it was possible to set various combinations of immune traits for LR and LW regardless of the applied mv GWAS method.

Briefly, the BN is a graphical representation of a probability distribution over a set of variables [61–63]. The conditional independence (of the random variables) and graphical separation (of the corresponding nodes of the graph) have been stretched out to disjoint node subsets by Pearl (1988). Therefore, in the BN approach model selection algorithms were used to learn the graphical structure of the network and then estimate the parameters of the local distribution functions conditional on the learned structure. A hill-climbing algorithm [28] was applied to the immune data set in this study. This Score-based structure learning algorithm is a general heuristic optimization technique to the problem of learning the structure of a BN. This algorithm attempts to maximize a score that measures how well that BN describes its goodness of fit to the data set, returning a graphical structure as output [63]. R package bnlearn [61] was used to obtain BNs for LR and LW immune trait residuals. Residuals of originally measured phenotypes were used to avoid a large number of solutions that need to be computed because of existing cross-classified effects. Resulting conditional dependencies illustrated as parents of the nodes in the network structure were used as trait combinations for mv GWAS approaches.

### Multivariate GWAS

GWAS is generally performed on a uv (trait-by-trait) basis by testing each variant at a time. Association analyses that include multiple phenotypes may be more powerful to identify QTL for complex traits, particularly in the case of causal variants that affect multiple correlated traits [64]. In the following, principles and optional parameters of four selected mv GWAS approaches applied in this study within each breed data set are described briefly.

#### Canonical correlation analysis

In the same way that PCA is applied to one set of possibly correlated traits to extract a number of independent variables (PCs) that explain as much variance in the original data set, CCA is applied to two sets of variables to extract a number of independent pairs of variables that explain as much covariance between the two original sets [65]. Thus, CCA represents a mv generalization of the Pearson product-moment correlation [66]. CCA extracts the linear combination of traits that explain the largest possible amount of the covariation between the marker and all traits. This approach is applied to analyze the association between one SNP and multiple traits, as implemented in --mqfam --mult-pheno procedure for MV-PLINK [65]. The test implies Wilk’s lambda (λ) and the corresponding F-approximation. Specifically, $\lambda =1-{\stackrel{\prime }{\rho }}^{2}$, where $\stackrel{\prime }{\rho }$ is the canonical correlation between the marker and the traits, calculated as the square root of the eigenvalue of the product of the marker variance (${S}_{11}$), trait covariance matrix (${S}_{22}$), and covariance matrices between the marker and the traits (${S}_{12}{,S}_{21}$); expressed as notation: ${S}_{11}^{-1/2}\times {S}_{12}\times {S}_{22}^{-1}\times {S}_{11}^{-1/2}$ [65]. Similar to PCA, an asymptotic significance mv p-value is generated in the CCA output. This p-value was subsequently adjusted for population stratification and multiple testing on the genome and chromosome levels.

#### Meta-analysis

Methodology development to increase the statistical power of GWAS is extremely important for study designs with heterogeneous traits and small sample sizes. Meta-analysis was carried out with the software TATES (Trait-based Association Test that uses Extended Simes procedure) [67]. TATES requires a phenotype correlation matrix of immune traits and a list of p-values in an ascending order of the phenotypes for a given SNP obtained in a corresponding uv linear regression analysis. During a meta-analysis uv GWAS was performed for each phenotype with PLINK [57]. Obtained p-values were adjusted to account for multiple testing and relationships between immune traits within the meta-analysis on the genome level. TATES combines the phenotype-specific p-values to obtain one overall trait-based p-value $\left({P}_{T}\right)$ as ${P}_{T}=Min\frac{{m}_{e}{p}_{j}}{{m}_{ej}}$, where ${m}_{e}$ indicates the effective number of independent p-values of all phenotypes, and ${m}_{ej}$ is the effective number of p-values among the top p-values, and ${p}_{j}$ is the j^th^ p-value [67]. Based on the procedure developed by Li et al. [68], the effective number of p-values (${m}_{ej}$is estimated through a correction based on eigenvalue decomposition of the correlation matrix between the p-values associated with the phenotypes. Briefly, TATES transforms the trait correlation matrix into a corresponding SNP-p-value correlation matrix. The eigen-decomposition of this p-value correlation matrix is used to weight uv p-values. Finally, the minimum of these weighted p-values is chosen as the corrected p-value for the joint association.

#### Bayesian multivariate regression

With the software mvBIMBAM (multivariate Bayesian IMputation-Based Association Mapping) [69] a Bayesian multivariate regression test for association was conducted. Simultaneously the traits were subdivided according to their SNP effect and Bayes Factors were used to access the association between the groups of phenotypes and a genetic variant. The analysis is based on the mv regression model like model (1), but with a $Y$(n x d) matrix of d phenotypes measured on each of n individuals. The mvBIMBAM approach attempts to partition the response variables Y into three groups according to their statistical association with a genetic variant: undirect (U), direct (D), and indirect (I). A set of models γ = (U, D, I) runs through partitions of the coordinates {1; …, d}. Under model γ an assumption is made that Y_U_ is independent of Z, and Y_I_ is conditionally independent of Z given Y_D_. This gives

$${P}_{\gamma }={P}_{\gamma }\left({Y}_{U}\right){P}_{\gamma }\left({Y}_{D}\vee {Y}_{U},Z\right){P}_{\gamma }\left({Y}_{I}\vee {Y}_{U},{Y}_{D}\right)$$

These scenarios were accessed with the option mph 2 within the mvBIMBAM software. The priors for the genetic effect were set at 0.1 and 0.2 according to the author’s recommendation [69]. Bayes Factor is computed as the support for partition γ compared with the global null hypothesis that all the phenotypes are unassociated with Z. It then summarizes the overall evidence against the null, as well as the posterior probability that each coordinate of Y is associated with Z:

$${BF}_{\gamma }=\frac{{P}_{\gamma }\left(Y\vee Z\right)}{{P}_{0}\left(Y\right)}$$

Obtained log_10_ Bayes Factors for each genetic variant evaluated the association between the SNP and the traits averaging over all possible partitions. Log_10_ Bayes Factors value ≥ 3 was characterized as a spurious association while values ≥ 6 as a solid association between a marker and a trait on genome level.

### Controlling population stratification and false-positive results

Genomic Control [70] was realized to correct for existing population stratification through adjustment of the significance of the test statistic in R [71]. From GWAS obtained p-value was subsequently adjusted in the PCA and CCA. The inflation factor lambda was low to moderate in the LR (0.80–1.26) and LW (0.86–1.23) data sets. After stratification correction, the lambda values were acceptable in a range of < 1.05.

To control the number of false-positive results False Discovery Rate (FDR) was applied [72] on genome and chromosome level for uv linear regression method, PCA, and CCA. The significance level q (p-values adjusted with FDR) for FDR was 0.05 to detect associations between marker and trait on genome and chromosome level in R [71]. Bayesian approaches express significance with a log10 Bayes Factor threshold. Absolute values of three and six are considered as spurious and solid significance for an association [32].

For uv and mv GWAS QTL regions were defined considering significant SNPs that mapped at least ± 1 Mbp from another significant SNP and functional annotation was performed retrieving all annotated genes within a QTL region in *Sus scrofa*11.1 from Ensembl database [49].

### Electronic supplementary material

Below is the link to the electronic supplementary material.

Supplementary Material 1

Supplementary Material 2

Supplementary Material 3

Supplementary Material 4

## Data Availability

Data cannot be made publicly available, as they are owned by a third party, the BHZP GmbH. The datasets used and analyzed during the current study are available from the corresponding author on reasonable request and with permission of the BHZP GmbH pig breeding company (henne@bhzp.de).

## Abbreviations

AAMP: Angio associated migratory cell protein
AGT: Aangiotensinogen
APP: Acute-Phase-Protein
BAS: Basophils
BIMBAM: Bayesian linear regression uv approach
CCA: Canonical correlation analysis
EOS: Eosinophils
FDR: False Discovery Rate
GWAS: Genome-wide association study
HAP: Haptoglobin
HMG: Hemoglobin
HMT: Hematocrit
HWE: Hardy-Weinberg equilibrium
IBS: Identity-by-state
IFN: Interferone
IL: Interleukin
LR: Landrace
LW: Large White
LYM: Lymphocytes
MAF: Minor allele frequency
MCH: Mean Corpuscular Hemoglobin
MCHC: Mean Corpuscular Hemoglobin Concentration
MCV: Mean Corpuscular Volume
MON: Monocytes
mv: Multivariate
mvBIMBAM: mv Bayesian linear regression approach/ mv Bayesian IMputation-Based Association Mapping
PC2Cyto: Second PC in the biological functional network cytokines
PCA: Principal component analysis
PNDK: Paroxysmal nonkinesiogenic dyskinesia domain containing
QTL: Quantitative Trait Loci
RBC: Red blood cells
r_g_: Genetic correlation
SNPs: Single Nucleotide Polymorphisms
TATES: Meta analysis/ Trait-based Association Test that uses Extended Simes procedure
TLL1: Tolloid like 1
TMBIM1: Transmembrane BAX inhibitor motif containing 1
TNF: Tumor Necrosis Factor-α
uv: Univariate
