# Supplementary material for: Multivariate genome-wide associations for immune traits in two maternal pig lines
Source: BMC Genomics. 2023 Aug 28;24:492. doi: 10.1186/s12864-023-09594-w (PMC10463314; doi:10.1186/s12864-023-09594-w)
Supplement: Supplementary file 1 — Supplementary Material 1 [file 12864_2023_9594_MOESM1_ESM.docx]

## Additional Files

**Additional Figure S1 - Comparison of different methods used to detect significant univariate associations for Landrace and Large White (.tiff)**

Multiple identical significant SNPs for different immune traits within a method are counted a single time. uv=univariate, LR=Landrace, LW=Large White.

**Additional Table S1 - Significant associated genetic markers identified with univariate methods (.xlsx)**

SSC=Sus scrofa chromosome, SNP=single nucleotide polymorphism, m/M allele=minor/major allele, MAF=minor allele frequency, QTL nr.=Quantitative trait loci progressive number based on ±1Mbp distance from a significant SNP, LR=Landrace, LW=Large White, RBC=Red blood cells, HMG=Hemoglobin, HMT=Hematocrit, MCV= Mean Corpuscular Volume, MCH=Mean Corpuscular Hemoglobin, MCHC=Mean Corpuscular Hemoglobin Concentration, PLT=Platelets, WBC=White blood cells, NEU=Neutrophils, LYM=Lymphocytes, MON=Monocytes, EOS=Eosinophils, BAS=Basophils, HAP=Haptoglobin, IFN-γ= Interferon-γ, IL=Interleukin, TNF-α= Tumor Necrosis Factor-α.

**Additional Table S2 - Significant associated genetic markers identified with multivariate methods (.xlsx)**

SSC=*Sus scrofa* chromosome, SNP=single nucleotide polymorphism, m/M allele=minor/major allele, MAF=minor allele frequency, QTL nr.=Quantitative trait loci progressive number based on ±1Mbp distance from a significant SNP, LR=Landrace, LW=Large White, RBC=Red blood cells, HMG=Hemoglobin, HMT=Hematocrit, MCV= Mean Corpuscular Volume, MCH=Mean Corpuscular Hemoglobin, MCHC=Mean Corpuscular Hemoglobin Concentration, PLT=Platelets, WBC=White blood cells, NEU=Neutrophils, LYM=Lymphocytes, MON=Monocytes, EOS=Eosinophils, BAS=Basophils, HAP=Haptoglobin, IFN-γ= Interferon-γ, IL=Interleukin, TNF-α= Tumor Necrosis Factor-α, PC=Principal Component, CCA=Canonical Correlation Analysis, PCA=Principal Component Analysis.
