# Supplementary figures and images for: Multivariate genome-wide associations for immune traits in two maternal pig lines
Source: BMC Genomics. 2023 Aug 28;24:492. doi: 10.1186/s12864-023-09594-w (PMC10463314; doi:10.1186/s12864-023-09594-w)

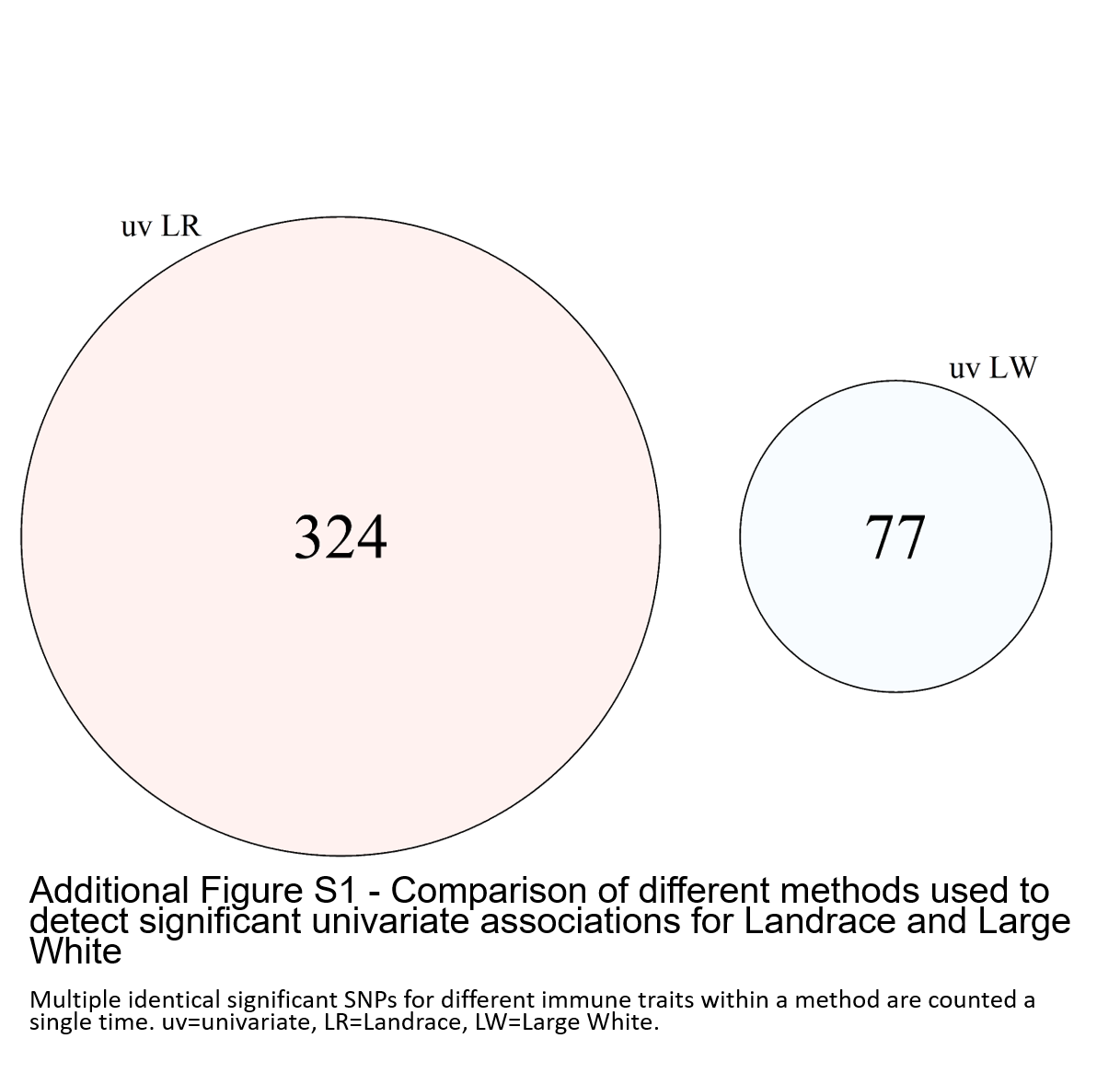

Supplement: Supplementary file 2 — Supplementary Material 2 [file 12864_2023_9594_MOESM2_ESM.tiff]
